# Attention is complex: causes and effects

**DOI:** 10.3389/fpsyg.2015.00246

**Published:** 2015-03-12

**Authors:** Olivier A. Coubard

**Affiliations:** ^1^The Neuropsychological Laboratory, CNS-FedParis, France; ^2^Laboratoire Psychologie de la Perception, UMR 8242 CNRS-Université Paris DescartesParis, France

**Keywords:** attention, selection, basal ganglia, superior colliculus, theory

Recently, Krauzlis et al. (2014) introduced a framework in which attention arises from value-based decision-making mechanisms centered on basal ganglia. Appropriate decision-making is made by identifying the current “state” of the animal and its environment. Defining a state requires interpreting different sources of information from the subject's internal and external world, knowledge and needs. The authors provide evidence from physiology, neuroanatomy, clinical studies and modeling. Here I re-examine this framework in the light of the following keys that help understand a cognitive function: its architecture and neural correlates, its process and mechanisms, its theory and metaphors. The component of attention described by Krauzlis et al. arises as a functional consequence of basal ganglia activity, suggesting that it may be treated as an effect not a cause (Krauzlis et al., 2014).

Since the original work by James (1890/1950), thousands of contributions have been published in the field of attention (e.g., over 59791 articles in PudMed with “attention” as a subheading). From this ocean of research, some consensus has emerged about the *architecture* of attention. In psychology, three attention macrosystems have been delineated: arousal or vigilance, selective attention or selection, and attentional control (Pashler, 1998). These modules have been validated by physiology as corresponding to distinct neural networks of the animal (Maunsell, 2004) and of the human (Parasuraman, 2000) brains. Vigilance has been associated with the activity of brainstem reticular formation and diffuse thalamic substance (Robbins, 1997; Coull, 1998; Paus, 2000); selection with that of cortical—particularly parietal—and subcortical areas (Snow et al., 2009; Lovejoy and Krauzlis, 2010; Capotosto et al., 2013); and control with that of cortical—particularly prefrontal—and subcortical areas (Desimone et al., 1990; Knight et al., 1995; Stuss, 2006). In light of this architecture, I suggest that Krauzlis et al. (2014)'s model might not concern attention as a whole but may be restricted to *one* attentional system: selection and even more spatial selection. My argument is twofold. First, 32 of their 102 references (n°1, 2, 3, 4, 5, 6, 7, 8, 11, 12, 13, 14, 15, 16, 24, 33, 44, 52, 53, 54, 55, 56, 58, 59, 60, 64, 65, 66, 67, 68, 75, 100) are dedicated to selection whereas 0 and 1 (n°81) respectively concern vigilance and control. Second, the reported neural bases and related brain damages are compatible with those of selection and related disorders: spatial neglect and Parkinson's disease (Krauzlis et al., 2014).

With respect to the *process*, the macromechanism regulating attention uses subcortical pathways. First, Krauzlis et al. suggest that the route from superior colliculus (SC) to medial dorsal nucleus of thalamus provides corollary discharge signals about eye movements to frontal eye field and convey signals to the striatum through prefrontal cortex. Second, the route from SC intermediate layers to the parafascicular nucleus through thalamus would be the predominant source of thalamus inputs to pathways in the striatum. Third, the route from SC intermediate layers to substantia nigra pars compacta would provide signal related to the detection of salient sensory events. Other routes leading to basal ganglia may play a role in this non-cortical circuit for attention (Krauzlis et al., 2014). It is a quality of the authors to rehabilitate the subcortex in (selective) attention and to recall that attention is not the exclusivity of cortex. They indeed review that invertebrates (honeybees, *Drosophilia*) or vertebrates without cortices (pigeons, peacocks, owls, frogs, salamanders or zebrafish) do exhibit signs of attention. Such model is reminiscent with (pre)motor theories of attention highlighting the role of subcortical areas in attention—whether it be called attention, imagination, emulation, simulation or projection. Nineteen years ago, Berthoz (1996) suggested the role of areas from brainstem to basal ganglia in imagination as a functional consequence of movement gating, and Kustov and Robinson (1996) demonstrated the critical role of SC in attention shifts. At the cell level, the micromechanism by which basal ganglia inputs are differentially weighted uses a process similar to Bayesian inference (Krauzlis et al., 2014). In this way, the model partially resembles competition models, whether they be called diffusion (Ratcliff, 1978), linear approach to threshold with ergodic rate (Carpenter, 1981) or race (Schall, 1995).

With regard to the *theory*, given that alternatively “everyone” (James, 1890/1950) and “no one” (Pashler, 1998) knows what attention is, the history of psychology has exploited different metaphors to account for what attention can be. The debates have turned around early vs. late filtering (Treisman, 1969), excitatory vs. inhibitory mechanisms (Laberge and Brown, 1989), or bottom-up (i.e., stimulus-driven or exogenous) vs. top-down (i.e., goal-directed or endogenous) processes (Awh et al., 2012). The cause vs. effect nature of attention has been reviewed by Fernandez-Duque and Johnson (2002), and the idea that attention may be an effect not a cause has been advanced by Desimone and Duncan (1995). In that sense, attention is an emergent property or epiphenomenon of the fact that when stimulus representations compete for processing resources, one of them wins (Fernandez-Duque and Johnson, 2002). In Krauzlis et al. (2014)'s model, attention is a functional consequence of the competition between basal ganglia circuits. Like other competition models, it has the advantage of banishing the control homunculus (Monsell and Driver, 2000) and the disadvantage of seeing attention as something we can do without (Fernandez-Duque and Johnson, 2002). But Krauzlis et al. (2014) also admit that control is still needed. Indeed they indicate that one function of inputs from prefrontal cortex to striatum could be to provide additional non-sensory inputs to expand the number of states and associated value functions that can be acquired. For that reason, I suggest that Krauzlis et al. (2014)'s model is likely to be a “biased competition model” according to Fernandez-Duque and Johnson (2002)'s taxonomy. Though the concept of a central executive is eliminated, the model is still using feedback loops that bias the information processing of upcoming stimuli. However the question of how such modulation gets decided remains a mystery. This leads me to the conclusion that Krauzlis et al. (2014)'s model does not prove attention to be mainly an effect. Rather their model may be a hybrid theory in which attention is complex, comprising causes and effects, where there is still room for attention as a cause.

## Conflict of interest statement

The author declares that the research was conducted in the absence of any commercial or financial relationships that could be construed as a potential conflict of interest.
